# Supplementary material for: Disentangling cephalopod chromatophores motor units with computer vision
Source: eLife. 2026 Jul 8;15:RP110074. doi: 10.7554/eLife.110074 (PMC13345625; doi:10.7554/eLife.110074)
Supplement: Peer review history

# Editor report: eLife Assessment

**DOI:** 10.7554/eLife.110074.3.sa0

**Authors:** John C Tuthill

**Affiliations:** https://ror.org/00cvxb145 University of Washington United States

**Keywords:** Convincing, Valuable

This **valuable** study uses a computer vision pipeline to infer the motor control of cephalopod skin, revealing that individual chromatophores exhibit anisotropic deformations and can be associated with multiple putative motor units. The evidence supporting these claims is **convincing**, and the authors present some limited electrophysiological validation of the findings from their computational analysis. This work will be of significant interest to biologists studying cephalopod behavior and motor control.

# Referee report: Reviewer #1 (Public review)

**DOI:** 10.7554/eLife.110074.3.sa1

Renard, Ukrow et al. applied their recently published computational pipeline (CHROMAS) to the skin of Euprymna berryi and Sepia officinalis to track the dynamics of cephalopod chromatophore expansion. By segmenting each chromatophore into radial slices, and analyzing the co-expansion of slices across regions of the skin, they inferred the motor control underlying chromatophore groups.

Strengths:

- The authors demonstrate that most motor units of cephalopod skin include a subregion of multiple chromatophores, creating "virtual chromatophores" between fixed chromatophores. This is an interesting concept that challenges prevailing models of chromatophore organization, and raises interesting possibilities for how chromatophore arrays may be patterned during development.

- This study introduces new analytical approaches of cephalopod skin that will be valuable for the quantitative study of cephalopod behavior.

Weaknesses:

- The authors use patch-clamp experiments in E. berryi to test their approach for inferring motor units. The stimulations indeed evoke expansions of sub-regions of each chromatophore, creating "virtual chromatophores". However, they were not able to predict these motor units from behavioral analysis before confirming them with patch-clamp, limiting the strength of this validation.

- In S. officinalis, chromatophores are far more numerous than in E. berryi and exhibit frequent spontaneous activity, making it more challenging to distinguish shared motor drive. Patch-clamp experiments in this species would provide important validation and strengthen confidence in the method for inferring motor units.

- Although multiple experimental conditions were tested (e.g., age, size, behavioral context, sedation, head-fixation, lighting), data is only shown from a small subset of experiments. Analyzing pooled data across conditions would allow for more generalizable conclusions.

- Different clustering algorithms were used for the two species (HDBSCAN for E. berryi and Affinity Propagation for S. officinalis). Since Affinity Propagation appeared to better capture correlation structure in S. officinalis, it would be informative to reanalyze the E. berryi data using the same method to assess potential algorithm-dependent biases.

Conclusion:

The CHROMAS tool is likely to be valuable to the field, given the need for quantitative frameworks in cephalopod biology. The predictions outlined here provide a useful foundation for future experimental investigation.

# Referee report: Reviewer #2 (Public review)

**DOI:** 10.7554/eLife.110074.3.sa2

Summary:

Overall, this is an excellent paper, making use of a newly developed system for monitoring the behaviour of chromatophores in the skin of (mostly) free swimming bobtail squid and European cuttlefish. The manuscript is very well written, clearly presented and very well structured. The central finding, that individual chromatophores are connected to multiple motor neurones, is not new. Novelty instead comes from the ability to measure the actuation of chromatophore sections across wide areas of skin in free-swimming animals, showing the diversity of local motor units and reinforcing the notion that individual chromatophores are not necessarily the individual units of colour change, but rather local motor units that cover multiple neighbour and near neighbour chromatophore muscles. This is an excellent finding and one that will shape our understanding of the neural control of cephalopod skin colour. I have a number of minor points below that the authors will need to address before acceptance.

Strengths:

The methodological approach to collecting large amounts of data about local variations in the expansion of sections of chromatophores is exciting, and the analysis pipeline for clustering sections of chromatophores whose spontaneous activity correlated over time is powerful and exciting.

Comments on revisions:

All concerns have been addressed in the revised version of the manuscript.

# Referee report: Reviewer #3 (Public review)

**DOI:** 10.7554/eLife.110074.3.sa3

Summary:

This study uses high-resolution videography and a custom computer-vision pipeline to dissect the motor control of cephalopod chromatophores in Euprymna berryi and Sepia officinalis. By quantifying anisotropic chromatophore deformations and applying dimensionality reduction methods, the authors infer that individual chromatophores can be a part of multiple motor units. Clustering analyses reveal putative motor units that often span multiple chromatophores, with diverse and overlapping geometries. Chromatophore expansion dynamics are faster and more stereotyped than relaxation, consistent with active neural contraction followed by passive recoil. Together, the results show that chromatophores function not as uniform pixels but as fractionated, coordinately controlled elements that enable flexible pattern generation

Strengths:

The authors present compelling, direct evidence that (a). chromatophore deformations are anisotropic, and indirect evidence that (b). individual chromatophores can be split across multiple putative motor units. This evidence is provided through data collected over large spatial scales, but also at a sub-chromatophore resolution. This combination of scale and resolution is not possible using traditional neuroanatomical and physiological approaches alone.

The authors also develop a new non-invasive, image analysis approach to extract information about chromatophore deformation across large spatial scales on the organism's body. In principle this approach is applicable across species and may allow for further comparative characterization of chromatophore motor control. It is therefore a promising new tool and useful resource for the community.

Weaknesses:

An important weakness of the work is that the methods the authors develop can only be applied during resting, spontaneous 'flickering' activity of chromatophores to yield interpretable results at the motor unit level. This is because common presynaptic input would confound the identification of individual motor units. Thus, there remains a large difficulty in linking insights about single motor unit organization to the circuit and behavioral levels.

Another weakness of this paper is the rather limited electrophysiological validation of the computational findings. The authors present only one electrophysiology experiment in E. berryi, the species that they used only for 'methodological development' and not for detailed characterization. A complementary electrophysiological experiment in S. officinalis, or some visualization of neuron morphology confirming that motor neurons do indeed project to multiple chromatophores would strengthen the generalizability of their computational analysis. This would be particularly pertinent to validate the author's claim that some motor units contain chromatophores that are quite distant from one another on the animal.

Overall, the authors' technical contributions and method development are an important advance. This work serves as an excellent proof of concept that their method can extract useful information about chromatophore motor control. Further validation of their method is needed to fully trust the fine-scale conclusions drawn about the distribution and composition of multi-innervated chromatophores. Furthermore, the authors raise many interesting ideas about developmental constraints on circuit wiring and potential adaptive significance of multi-innervated chromatophores for certain features of camouflage patterning. Their method may be able to help resolve some of these questions in the future if it is refined and applied across developmental stages, regions on the animal, and across species

Comments on revisions:

Thank you for clarifying my major point of confusion regarding how one might connect these results to behaviorally relevant camouflage. I now have a better understanding of the author's rationale in studying resting activity of motor units and believe that the clarifications added to the manuscript will help other readers who encounter similar confusion.

# Author comment: Author response

**DOI:** 10.7554/eLife.110074.3.sa4

**Authors:** Gilles Laurent, Mathieu DM Renard, Johann Ukrow, Margot Elmaleh, Dominic A Evans, Yifan Wu, Xitong Liang

**Affiliations:** https://ror.org/02h1nk258 Max Planck Institute for Brain Research Frankfurt Germany; https://ror.org/02h1nk258 Max Planck Institute for Brain Research Frankfurt Germany; https://ror.org/02h1nk258 Max Planck Institute for Brain Research Frankfurt Germany; https://ror.org/02h1nk258 Max Planck Institute for Brain Research Frankfurt am Main Germany; https://ror.org/02h1nk258 Max Planck Institute for Brain Research Frankfurt am Main Germany; https://ror.org/02v51f717 Peking University Beijing China; https://ror.org/02v51f717 Peking University Beijing China

The following is the authors’ response to the original reviews.

**Public Reviews:**

**Reviewer #1 (Public review):**

Summary:

Renard, Ukrow et al. applied their recently published computational pipeline (CHROMAS) to the skin of Euprymna berryi and Sepia officinalis to track the dynamics of cephalopod chromatophore expansion. By segmenting each chromatophore into radial slices and analyzing the co-expansion of slices across regions of the skin, they inferred the motor control underlying chromatophore groups.

Strengths:

The authors demonstrate that most motor units of cephalopod skin include a subregion of multiple chromatophores, creating "virtual chromatophores" in between the fixed chromatophores. This is an interesting concept that challenges prevailing models of chromatophore organization, and raises interesting possibilities for how chromatophore arrays may be patterned during development.

This study introduces new analyses of cephalopod skin that will be valuable for the quantitative study of cephalopod behavior.

Weaknesses:

The authors chose to image spontaneous skin changes in sedated animals, rather than visually-evoked skin changes in awake, freely-moving animals. Spontaneous chromatophore changes tend to be small shimmers of expansion and contraction, rather than obvious, sizable expansions. This may make it more challenging to distinguish truly co-occurring expansions from background activity. The authors don't provide any raw data (videos) of the skin, so it is difficult to independently assess the robustness of the inferred chromatophore groupings.

The patch-clamp experiments in E. berryi are used to test the validity of their approach for inferring motor units. The stimulations evoke expansions of sub-regions of each chromatophore, creating "virtual chromatophores" as predicted from the behavioral analysis. However, the authors were not able to predict these specific motor units from behavioral analysis before confirming them with patch-clamp, limiting the strength of the validation. It would be informative to quantify the results of the patch-clamp experiments - are the inferred motor units of similar sizes to those predicted from behavior?

The authors report testing multiple experimental conditions (e.g., age, size, behavioral stimuli, sedation, head-fixation, and lighting), but only a small subset of these data are presented. It is difficult to determine which conditions were used for which experiments, and the manuscript would benefit from pooling data from multiple experiments to draw general conclusions about the motor control of cephalopod skin.

The authors use a different clustering algorithm for E. berryi and S. officinalis, but do not discuss why different clustering approaches were required for the two species.

Impact:

The authors use their computational pipeline to generate a number of interesting predictions about chromatophore control, including motor unit size, their spatial distribution within the skin, and the independent control of subregions within individual chromatophores by putatively distinct motor neurons. While these observations are interesting, the current data do not yet fully support them.

The CHROMAS tool is likely to be valuable to the field, given the need for quantitative frameworks in cephalopod biology. The predictions outlined here provide a useful foundation for future experimental investigation.

We thank the reviewer for the thoughtful and detailed evaluation of our work and for recognizing the potential of the CHROMAS pipeline for studying chromatophore control.

We agree that some aspects of the manuscript required clarification and additional explanation, and we have revised the text accordingly. We also now provide access to representative raw video recordings in the Data Availability section. In the *E. berryi* patch-clamp experiments, single motor neurons evoked expansions of sub-regions of chromatophores, consistent with the “virtual chromatophore” concept. We have now quantified the size of motor units across patch-clamp sessions, and the results show that the inferred motor-unit sizes broadly match those predicted from behavioral recordings, supporting the validity of our approach.

We agree that pooling data across individuals would provide valuable insight into variability across animals. In practice, we recorded chromatophore activity from several animals (14 *Euprymna berryi* and 12 *Sepia officinalis*) under different experimental conditions during development of the experimental pipeline. However, acquiring long, stable, artifact-free recordings suitable for motor unit analysis is technically challenging. We now clarify this point in the manuscript. Specifically, we explain that multiple animals were recorded during pipeline development, while the analyses presented focus on recordings with the highest signal quality. We anticipate that the framework introduced here will enable future studies to collect larger datasets and compare motor unit organization across individuals, developmental stages, and species.

HDBSCAN was used for *E. berryi* during initial exploratory analyses, and Affinity Propagation was adopted for *S. officinalis* because it better captured the correlation structure of those recordings. We did not re-analyze the *E. berryi* data with Affinity Propagation, and the implications of algorithm choice are now discussed in the Discussion.

**Reviewer #2 (Public review):**

Summary:

Overall, this is an excellent paper, making use of a newly developed system for monitoring the behaviour of chromatophores in the skin of (mostly) free-swimming bobtail squid and European cuttlefish. The manuscript is very well-written, clearly presented and very well-structured. The central finding, that individual chromatophores are connected to multiple motor neurones, is not new. Novelty instead comes from the ability to measure the actuation of chromatophore sections across wide areas of skin in free-swimming animals, showing the diversity of local motor units and reinforcing the notion that individual chromatophores are not necessarily the individual units of colour change, but rather local motor units that cover multiple neighbour and near-neighbour chromatophore muscles. This is an excellent finding and one that will shape our understanding of the neural control of cephalopod skin colour.

Strengths:

The methodological approach to collecting large amounts of data about local variations in the expansion of sections of chromatophores is exciting, and the analysis pipeline for clustering sections of chromatophores whose spontaneous activity correlated over time is powerful and exciting.

Weaknesses:

Some minor edits and typographical errors need correcting. I also had some concerns that the preparation for the electrophysiological section of the manuscript complies with the journal's ethical requirements, so I would urge that this be carefully checked.

We thank the reviewer for the positive evaluation of our work and for recognizing the value of the methodological approach and the clarity of the manuscript.

We have carefully reviewed the manuscript and corrected minor typographical errors.

Regarding the ethical considerations raised for the electrophysiological experiments, we have carefully verified that the experimental procedures comply with the journal's ethical requirements and relevant institutional guidelines.

**Reviewer #3 (Public review):**

Summary:

This study uses high-resolution videography and a custom computer-vision pipeline to dissect the motor control of cephalopod chromatophores in Euprymna berryi and Sepia officinalis. By quantifying anisotropic chromatophore deformations and applying dimensionality reduction methods, the authors infer that individual chromatophores can be a part of multiple motor units. Clustering analyses reveal putative motor units that often span multiple chromatophores, with diverse and overlapping geometries. Chromatophore expansion dynamics are faster and more stereotyped than relaxation, consistent with active neural contraction followed by passive recoil. Together, the results show that chromatophores function not as uniform pixels but as fractionated, coordinately controlled elements that enable flexible pattern generation

Strengths:

The authors present compelling, direct evidence that (a). chromatophore deformations are anisotropic, and indirect evidence that (b) individual chromatophores can be split across multiple putative motor units. This evidence is provided through data collected over large spatial scales, but also at a sub-chromatophore resolution. This combination of scale and resolution is not possible using traditional neuroanatomical and physiological approaches alone.

The authors also develop a new non-invasive, image analysis approach to extract information about chromatophore deformation across large spatial scales on the organism's body. In principle, this approach is applicable across species and may allow for further comparative characterization of chromatophore motor control. It is therefore a promising new tool and useful resource for the community.

Weaknesses:

An important weakness of the work is that the methods the authors develop can only be applied during resting, spontaneous 'flickering' activity of chromatophores. The inability to reliably apply their technique during any kind of realistic camouflage is a large limitation, as it means this method cannot be used to study the dynamics of motor control during realistic camouflage behaviors.

Another weakness of this paper is the rather limited electrophysiological validation of the computational findings. The authors present only one electrophysiology experiment in E. berryi, the species that they used only for 'methodological development' and not for detailed characterization. A complementary electrophysiological experiment in S. officinalis, or some visualization of neuron morphology confirming that motor neurons do indeed project to multiple chromatophores, would strengthen the generalizability of their computational analysis. This would be particularly pertinent to validate the author's claim that some motor units contain chromatophores that are quite distant from one another on the animal.

Overall, the authors' technical contributions and method development are an important advance. This work serves as an excellent proof of concept that their method can extract useful information about chromatophore motor control. Further validation of their method is needed to fully trust the fine-scale conclusions drawn about the distribution and composition of multi-innervated chromatophores. Furthermore, the authors raise many interesting ideas about developmental constraints on circuit wiring and potential adaptive significance of multi-innervated chromatophores for certain features of camouflage patterning. Their method may be able to help resolve some of these questions in the future if it is refined and applied across developmental stages, regions of the animal, and across species

We thank the reviewer for their thoughtful evaluation and for recognizing the potential of the computational approach introduced in this study.

Regarding the focus on spontaneous chromatophore activity, we have clarified earlier in the Results section why these events are necessary to isolate individual muscle activations. While large camouflage patterns are visually striking, they involve the coordinated activation of many groups of chromatophores by premotor circuits simultaneously, making the identification of individual motor units, our goal here, impossible. Our approach can, however, also be applied during active behavior, including camouflage; the questions addressed there would be different, focusing on how multiple motor units are coordinated to generate the resulting skin patterns, rather than resolving the structure of single motor units. This could be challenging if the patterns of premotor control are highly variable, thus making the detection of meaningful or interpretable motion correlations difficult. This remains to be tested.

We also acknowledge that electrophysiological validation remains limited. Patch-clamp experiments were performed in *Euprymna berryi* to test predictions generated by the computational analysis, and these experiments confirmed that activation of single motor neurons can produce anisotropic expansion of chromatophore subregions. We now provide the associated datasets in the Data Availability section. We agree that complementary electrophysiological or anatomical experiments in *Sepia officinalis* would further strengthen the conclusions. Such experiments represent an important direction for future work.

**Recommendations for the authors:**

**Reviewer #1 (Recommendations for the authors):**

General points:

(1) Given all the experimental conditions and animals tested, the manuscript would be much stronger if the figures represented pooled data from many animals and experiments (e.g. Figure 1C).

We agree that pooling data from multiple animals would strengthen the manuscript. In practice, we tested these experimental conditions across several animals (14 *Euprymna berryi* and 12 *Sepia officinalis*), but we selected the segments shown in the figures for their minimal artifacts and errors. Acquiring high-quality, stable recordings of this type is extremely challenging, and the presented data represents the clearest examples suitable for analysis and visualization. We hope that in the future these methods will enable not only the collection of a larger, high-quality dataset, but also comparisons across individuals, ages, species, and different regions of the mantle.

(2) It's very unclear what animals were used for each experiment:

(a) E. berryi: L677 states that 14 animals were filmed, and L684 implies that non-sedated individuals were used in addition to sedated animals, but it appears all the data is from a single E. berryi with sedation?

The original wording was unclear, so we modified the sentence for clarity. The Methods now specify that 14 animals were filmed to refine the experimental pipeline and explore different conditions, while the data presented in the Results are from a single lightly sedated individual chosen for quality and stability of chromatophore activity.

(b) S. officinalis: L692 onwards states that lots of different conditions and animals were explored, but only minimal data from a couple of animals is described in the figures. L156 states that all (?) the data comes from one head-fixed animal and one sedated and head-fixed animal. L549: The conclusion states that the pipeline was used in freely moving animals, but it appears that all of the S. officinalis were head-fixed? This is very confusing. Rather than describing the conditions of every experiment ever performed, the manuscript would benefit from explicitly stating the experimental conditions used for each figure.

The original text was unclear. We have clarified in the manuscript which animals and experimental conditions were used for the analyses in each figure. To clarify, *E. berryi* was recorded without head fixation, whereas *S. officinalis* data were obtained under head-fixed conditions. We did film 11 *S. officinalis* without head fixation, and data can in principle be extracted from these recordings. Head fixation was used both to minimize visual artifacts and to enable longer, stable recordings, which was important for capturing the highest level of apparent noise in motor unit activation—information that is critical for our analyses of motor-unit organization, though not necessary for studies of broader camouflage patterns. Our computational pipeline enables large-scale analyses that would be very difficult or impossible with traditional electrophysiology, not that all data were acquired from freely behaving animals. While fully unconstrained recordings remain technically challenging due to optical and logistical constraints, we maintain that our approach provides a valid framework for analyzing freely behaving animals.

(c) Additionally, there is a claim that the sedated condition represents the unsedated one (e.g. L151 and L643), but no data is shown to support this. L173 references Figure 6d as evidence, but 6d doesn't exist. Only L210 provides sedation/no sedation statistics for the number of components per motor unit. However, in L643 it says "and motor unit organization remained unchanged". This data needs to be shown to include that statement.

Reference to the inexistant 6d figure was removed. L170 provides statistics for the number of principal components per chromatophore, and L210 provides statistics for the number of components per MU. We do not think a sub-figure is necessary. We, however, agree that L643 “motor unit organisation” is potentially misleading as we only compared the number of chromatophores belonging to a single MU and not the MU shape or distribution. Changed “organization” to “size (in chromatophores)”.

(3) The text needs considerable revision. There are many typos (including multiple instances of "refs" instead of the actual references being inserted). These issues make the manuscript much more difficult to evaluate.

Our apologies. We have now added the missing refs.

(4) It is not clear how convincing the chromatophore groups are. For instance, Figure 4h could alternatively be interpreted as a group of 5 chromatophores in a motor group that happen to co-vary with a sixth one at a great distance. Without seeing some of the raw data (videos), it's difficult to assess how convincing it is that these chromatophores belong to the same group. I recommend analyzing: when multiple chromatophores expand together, what is the likelihood that other chromatophores also happen to expand at the same time (given the frequency that they're all changing shape spontaneously)?

We appreciate the reviewer’s concern. Chromatophores are assigned to the same cluster because their activity, or that of their slices, covaries consistently over time. It is, of course, possible that what appears as a single motor unit may reflect two or more motor neurons acting simultaneously during the recording. Longer video segments increase confidence in the integrity of inferred motor units, but in the absence of a ground truth for motor unit spatial organization in this species at this age, it is difficult to quantify the likelihood that two motor units are being conflated. Raw video data is provided in the Data Availability section. We note, however, that most of the time motor units cannot be readily discerned by eye, because individual chromatophores and their constituent slices fluctuate continuously, and motor-unit correlations are subtle and distributed across multiple chromatophores.

(5) The rationale for focusing on spontaneous activity is introduced relatively late in the manuscript and would benefit from being stated earlier. Examples should be provided of what this looks like (as opposed to regular chromatophore expansion). It would be valuable to see measurements across many experiments of how expanded the chromatophores are - what is the change in surface area? And what is the frequency of expansion for each chromatophore?

Thank you for the remark. This is true. We have added a paragraph at the beginning of the Results section to clarify the rationale for focusing on spontaneous activity.

This section now reads:

“Because our primary aim was to describe the composition and coordination of chromatophore motor units, it was important to examine animals in the absence of the descending commands that occur during active behavior. Spontaneous activity, typically mild and “noisy” was thus ideal to enable measurements of the motion correlations between chromatophores that reflected shared motor neuron drive, rather than shared correlations due to upstream motor neuron groupings by premotor circuits.”

We added an example of video recording of spontaneous activity in our Data Availability section.

While quantifying expansion magnitude and frequency across experiments would indeed be valuable, these questions fall outside the primary focus of the present study, which centers on resolving motor unit organization. In the section “Dynamics of chromatophore expansion and contraction,” we analyze the speed of expansion and contraction to demonstrate that such kinetic features can be reliably detected with the temporal resolution of our video imaging approach. By isolating single muscle activations, we establish a methodological framework that can be used in future work to quantify expansion amplitude, rate of change and frequency across preparations.

(6) Chromatophore expansion was only measured in anesthetized E. berryi, and L679 states that chromatophore expansion was triggered by shining light on the skin. However, light-mediated chromatophore expansion may be mediated by a different mechanism, so chromatophore correlations do not necessarily reflect the underlying motor control.

We agree that there is, in principle, a theoretical risk of direct light-mediated activation of chromatophores. Yet, the kinetics of this light mediated activation are very different, and are the object of a separate, on-going investigation by our groups. In our experiments, the illumination was applied to the whole animal rather than locally to the skin, ensuring that all chromatophores and the eyes were exposed to the same light source. By transitioning from darkness to light, we created a window in which chromatophores were partially expanded—both fully contracted and fully expanded states would show little to no decorrelation. Within this window, we observed spontaneous fluctuations in chromatophore activity, which formed the basis for our correlation analyses. To our knowledge, direct light-mediated expansion of chromatophores has not been reported in *E. berryi* although it may exist there. Finally, the size, shape, and orientation of the inferred motor units align with electrophysiological evidence, supporting the validity of our motor unit inferences.

(7) Some figures might be better suited for the supplement. For instance, it's not clear what the significance of Figure 5 is (it's not currently sufficiently justified in the text).

We have clarified the purpose of Fig. 5 in both the Results and Discussion sections. In the Results, we now explain that events are separated by amplitude to show that expansion–contraction kinetics can be reliably measured across a full range of chromatophore events, validating the precision of our videographic approach. In the Discussion, we highlight that this precision allows measurement of radial muscle speeds and opens avenues to study chromatophore biomechanics, including the contributions of intertwined forces such as radial muscles, elastic pigment sacs, and intercellular coupling.

(8) Multiple chromatophores can belong to multiple clusters - this study reveals that this is because subsections of a chromatophore are controlled separately. But do the same sections (slices) of chromatophores ever belong to multiple clusters?

Yes, it is possible. Dubas (1985) used videographic recordings to show that the same chromatophore muscle fibers could be activated by stimulation of different nerve bundles, supporting Florey’s (1969) electrophysiological evidence for polyneuronal excitatory innervation. From Dubas: "Usually, different muscle fibres were recruited by each nerve but sometimes a single muscle fibre responded to stimulation of each nerve. Variations of the stimulus voltage also produced gradation of the amplitude of shortening of individual muscle fibres. This supports the evidence above for multiple innervation of single muscle fibres."

The petal-like distribution of motor-neuron influence shows overlapping territories, suggesting that some chromatophore sections may be influenced by multiple neurons. However, this overlap could arise from polyinnervation of individual muscles, the presence of gap junctions between muscles, or passive mechanical coupling due to the elastic properties of the pigment sac.

The petal-like distribution of motor-neuron influence shows overlapping territories, suggesting that some chromatophore sections may be influenced by multiple neurons. However, this overlap could arise from polyinnervation of individual muscles, the presence of gap junctions between muscles, or passive mechanical coupling due to the elastic properties of the pigment sac.

With the present approach, it is not possible to disentangle the relative contributions of these mechanisms, which will require targeted physiological or anatomical experiments. For this reason, we adopted a hard clustering approach for individual chromatophore slices.

(9) All time should be labeled in seconds, not in frames, and all distances should be measured in um or mm, not in pixels.

We chose to present figures in pixels and frames to reflect the native units of our recordings and analyses, which preserves fidelity and reproducibility of the computational pipeline. For biological interpretation, corresponding values are converted to µm in the main text, providing the relevant real-world scale. A scale for conversion is provided in the figure legend.

Specific comments:

(1) L36: I'm not sure the description of virtual chromatophores here is clear enough to make sense to a more general audience.

Addressed. We retained the concept of ‘virtual chromatophores’ in the abstract and added a brief clarifying phrase to indicate that these are functional groupings of adjacent chromatophore territories that act as single units.

(2) L50: "Rimmed by" - consider rephrasing.

Addressed. Replaced with “surrounded”.

(3) L64: "refs" - actual references aren't inserted. There are multiple other examples of this.

Addressed. Added missing references.

(4) L100: This section could use rewriting. Some of the text reads more like a figure legend.

Addressed. We have streamlined the main text to reduce redundancy with the figure legend.

(5) L101: Consider the opening sentence/s providing a more general introduction to the question and approach.

Addressed.

(6) L104: This implies that the data presented are from 14 animals of many ages. This is only relevant if the pooled data is analyzed and presented.

We agree that the original phrasing was ambiguous. We have modified the sentence for clarity, and explain in the Methods that 14 animals were filmed to refine the pipeline and explore experimental conditions, while the analyses shown are from a single animal.

(7) L111: HDBSCAN should be defined.

Addressed. The acronym has been expanded.

(8) L173: Figure 6D doesn't exist.

Addressed. Reference to the inexistent 6d figure was removed.

(9) L193: "excluding negative (contraction) phases" This phrase requires clarification.

Addressed. Added “see Methods” in the legend and added clarification on the reasoning in Methods.

(10) L204: Should explain why the switch to affinity-propagation clustering was made when a different method was used for E. berryi.

Addressed in discussion.

(11) Figure 3: I recommend including a diagram or image of a whole cuttlefish and showing what the corresponding imaging area was in relation to the animal so the reader gets an intuitive sense of scale.

Thank you. We have added a supplementary figure to give the reader a sense of scale.

(12) L221/Fig 3b: These colors are supposed to represent clusters of 3 to 5 chromatophores? The clusters look much bigger.

The figure shows clusters of 3 to 5 chromatophores, but many adjacent clusters were assigned the same color. We have changed the colors to remove this ambiguity.

(13) Figure 3c: This would be more powerful if it represented the combined data of many experiments to draw a general conclusion. Also, shouldn't these cluster sizes match those in 2e, e.g. they get as big as 40?

We assume the reviewer is referring to a comparison between Figures 3c and 2e. For visualization purposes, the graph in 3c was truncated to display over 90% of the data, which explains why the largest clusters appear smaller than in 2e. We modified the legend accordingly. We agree that the results would be strengthened by pooling data from additional experiments; however, acquiring high-quality, artifact-free recordings suitable for motor unit analysis is extremely challenging. We hope that our framework will enable future studies to extend this analysis.

(14) Figure 4: I would show some of these examples earlier, to give the reader an intuitive sense of the data and claims (though it doesn't need its own figure - provide a couple of examples, and the diagram of how much of the mantle you're sampling) then put the rest in the supplement, and include some videos too.

We agree that providing spatial context is important for readers to develop an intuitive understanding of the dataset. However, introducing examples of motor units earlier in the manuscript would, in our view, interrupt the logical progression of the Results, where motor unit identification builds on prior analyses. To address the reviewer’s concern, we have added a new supplementary figure (Fig. S1) illustrating the size and location of the sampled mantle region. In addition, we now provide representative videos in the Data Availability section to give readers direct visual access to the underlying dynamics.

(15) Figure 4f: Is the location of the split color in each dot accurate? It's surprising that each one is split down the middle, and the pink side is always on the right - this is unintuitive given where the motor neuron is likely to be located.

The dots and half dots represent the membership of a chromatophore to a particular cluster.

(16) Figure 5: I didn't find this figure sufficiently justified in the text. I would move this to the supplement.

Addressed in General point #7.

(17) L350: States that 12 animals were patched, but the data isn't shown. It's important to show all of this data (some of which can be in the supplement).

Addressed. We provided the data in the Data Availability Section.

(18) Figure 5: I would quantify how many chromatophores were in each motor group across all the recording sessions, and compare this to the equivalent behavioral analysis.

We assume the reviewer means Fig. 6. We calculated and stated the size of motor units across patching sessions.

(19) Figure 5c: I recommend labeling each panel with a different number so you can refer to specific data.

We assume the reviewer means Fig. 6c. We consider the figure layout clear enough to allow readers to follow the data without additional panel numbers.

(20) L379: Typo: repeat of "quantitative"

Addressed.

(21) L576: Salinity should be 33-36 ppt, not %

Addressed.

(22) L877: The salinity units are sg? That should be stated. Though I would use the same units for salinity throughout.

Addressed.

Overall, this work introduces a potentially valuable quantitative framework for studying chromatophore dynamics. Addressing the points above would substantially strengthen the manuscript and clarify the scope and support for its conclusions.

We thank the reviewer for these many helpful comments.

**Reviewer #2 (Recommendations for the authors):**

(1) Line 64 - missing references for chromatophore colour with age.

Addressed. Added missing refs.

(2) Line 64-65 - would be good to have a little more detail about what is meant by 'migrating through the skin'. Is this a lateral process, or depth in the skin?

Addressed. Changed “migrating in the thickness..” with “through the thickness..” to emphasize verticality.

(3) Line 72 - typo, should read '...individual and groups...'

Addressed.

(4) Remove 'In Fig 1, ...' from line 104.

Addressed.

(5) Figure 1 - It's unclear why some chromatophores are uncoloured with a red dot in the centre. Are these chromatophores that do not share a cluster with neighbours? If so, wouldn't it make more sense to colour the chromatophore with a unique colour of its own? Or, at the very least, make a note in the caption to indicate that all white chromatophores are not clustered with neighbours.

Segmented chromatophores are shown in white, with coloured slices highlighting cluster membership. Uncoloured slices represent outliers. Addressed in the figure legend.

(6) Line 119 - the concept of a 'closed virtual chromatophore' needs a few more words of explanation. The way I interpret the text as it is, is that the motor units driving colour change are not necessarily the individual chromatophores, but a motor region containing a mixture of whole and partial chromatophores innervated by the same motor neuron. If this is the case, a few extra words of description would help here to remove any ambiguity as I think this is an important concept for the paper.

Addressed. We added a sentence clarifying the concept.

(7) Line 173 - Figure 6d doesn't exist in the paper. Was a different panel intended? If so, please make sure to number the figures in order of appearance in the manuscript.

Reference to the inexistent figure 6d was removed.

(8) Figure 3b is very difficult to see. Perhaps consider lightening the background image. Please also indicate whether the individual colours refer to individual clusters. If this is the case, then some of these clusters look much larger than the 3-5 suggested in the caption.

This issue has been corrected.

(9) Line 210 - remove the bold type.

Addressed.

(10) Line 211 - please specify which 'two groups' you are referring to here. Presumably, this is anaesthetised and non-anaesthetised.

Addressed.

(11) I think that the text is missing any indication of the pixel sizes involved in extracting slice metrics, particularly from the S. officinalis data. It would be great to include some data on how many pixels span the radius of an expanded chromatophore. There is some small indication of this in Figure 2a, but a panel or two with details about the pixel size of S. officinalis chromatophores and their slices would be welcome. This would help with the judgment of the robustness of the resolution of the analysis. Looking at the y-axis in Figure 5a, there is some indication that the chromatophore radius is only 1 to 8 pixels. Is this the case?

Figure 5a doesn’t show chromatophore radius but instead the relative change in peak amplitude during an expansion event. At that point the chromatophore has likely a larger radius as you sum the baseline radius of the chromatophore + the size of the peak.

(12) Line 246-7 - reword this sentence to avoid referring to Figure 3d in the narrative. Include it in parentheses instead.

Addressed.

(13) Lines 408 and 409 - missing references.

Addressed.

(14) Line 576 - salinity should be reported in parts per thousand, not per cent.

Addressed.

(15) Line 593 - how were animals <50mm fed?

Animals smaller than 50 mm were fed Neomysis spp. or small Palaemonetes spp., as noted a few lines above the description for animals larger than 50 mm.

(16) Line 847 - typo - '...putative motor units' ramifications...'

Addressed.

(17) Line 854 - better to write out the [chrom_id, label] info as narrative text rather than using the variable names.

Addressed.

(18) Line 876 - two typos '...were reared in an artificial...'

Addressed.

(19) Line 877 - please use the same salinity metric as used in the earlier part of the methods.

Addressed.

(20) Section 898-910 - equipment details would ideally include the location of the company. E.g. (BX51W1, Olympus, Tokyo, Japan).

Addressed.

**Reviewer #3 (Recommendations for the authors):**

I am left with a number of questions that arise from the authors' work, some of which the authors themselves briefly mention in the technical limitations section.

(1) In relation to the first weakness, do the authors know if the recruitment patterns they identify are likely to be the same when octopi perform visually-mediated camouflage to their environment?

Thank you for this comment. We assume the reviewer is referring to *S. officinalis*. There seems to be a misunderstanding: our approach is designed to reveal the smallest independent functional units—motor units—that together generate skin patterns. The technique is fully applicable to an animal displaying camouflage, but the results would necessarily differ. Camouflage patterns are composed of relatively large shapes compared to individual motor units and arise from the coordinated activation of multiple units. Disentangling motor units requires decorrelated activity, whereas visually-evoked camouflage inherently drives correlated motor-unit activation by premotor control. To use an analogy, if our goal were to map the distribution and wiring of pixels on a screen, it would be more informative to broadcast a noise signal rather than display coherent images, as the noise produces decorrelated activity that allows the underlying structure to be resolved. We have clarified this important point in the early results section.

(2) The authors provide indirect evidence that motor neurons innervate multiple chromatophores. Can sets of radial muscles within a chromatophore be innervated by multiple motor neurons? Is there neuroanatomical evidence or experiments that could perhaps shed light on this?

Addressed above. Same question as #1(8).

(3) Are multi-innervated chromatophores evenly distributed across the octopus's body? For instance, could the authors compare chromatophore recruitment over multiple patches on the animal from multiple regions?

At present, we do not have sufficient data to quantitatively compare motor-unit structure or the distribution of multi-innervated chromatophores across different body regions of cuttlefish. However, we would not necessarily expect uniformity across the skin, as distinct body regions are associated with characteristic pattern elements (e.g., the white square on the central mantle or the thicker zebra stripes along the sides). It is therefore plausible that different motor-unit geometries and densities are differentially represented across regions to support these region-specific patterns. Future recordings spanning multiple patches and body locations will be required to test this question directly.

(4) Relatedly, is there any idea of whether chromatophore size or age corresponds with the number of motor units within a single chromatophore?

At present, our analyses are limited to single developmental time points, and we therefore cannot directly assess whether chromatophore size or age correlates with the number of motor neurons innervating an individual chromatophore. However, this is a question that our analysis framework is explicitly designed to address. Our custom pipeline, CHROMAS, (Ukrow, Renard et al., 2025) includes tools for longitudinal image alignment that allow chromatophores to be tracked within the same animal across development. Applying these scripts to developmental datasets enables future analyses linking chromatophore growth or age to changes in the motor innervation of single chromatophores.

I understand that a full resolution to the issues raised above may require substantial additional experiments. At a minimum, further discussion of these points with integration of existing literature would elevate the paper.
